# E-cigarette use, psychological distress, and daily activity participation among adults in Riyadh

**DOI:** 10.3389/fpsyt.2024.1362233

**Published:** 2024-04-12

**Authors:** Fenoon Abdullah Aljohani, Bakriah Yahyaa Alzubaidi, Reem Hamdan Al-Rafdan, Hanan Mutiq Alblawi, Rama Hani Alrehayan, Ghada Abdulrahman Alsenan, Hadeil Salman Almohaya, Mona Mohamed Taha

**Affiliations:** ^1^ Department of Respiratory Therapy, College of Applied Medical Sciences, King Saud Bin Abdulaziz University for Health Sciences, Jeddah, Saudi Arabia; ^2^ Department of Occupational Therapy, College of Applied Medical Sciences, King Saud Bin Abdulaziz University for Health Sciences, Jeddah, Saudi Arabia; ^3^ Department of Rehabilitation Sciences, College of Health and Rehabilitation Sciences, Princess Nourah bint Abdulrahman University, Riyadh, Saudi Arabia

**Keywords:** adult, daily living activities, e-cigarettes, psychological distress, smoking

## Abstract

**Objectives:**

The prevalence of e-cigarettes is significantly increasing among adults as an alternative method to tobacco smoking. However, the chemical products of e-cigarettes have an influence on human general health. Therefore, this study aims to investigate the association between e-cigarette use and psychological distress as well as participation in daily activities among adults in Riyadh, Saudi Arabia. It also evaluates the demographic profile and usage patterns of e-cigarette users.

**Methods:**

This cross-sectional study involved 396 e-cigarette smokers in Riyadh city. An online survey was administered, including questions about smoking patterns and the perceived effects of e-cigarettes on activities of daily living. Additionally, mental health were assessed using the Kessler 6 scale.

**Results:**

Most of the participants were educated young males (61.4%). About 29.5% of the participants reported using e-cigarettes primarily to quit tobacco cigarettes. In addition, e-cigarette usage was significantly associated with lower participation in activities of daily living and higher psychological distress.

**Conclusion:**

This study found that many e-cigarette users are well-educated young individuals who use e-cigarettes as a substitute for traditional cigarettes. However, the study also revealed that using e-cigarettes is associated with negative effects on smokers’ daily activities and mental health.

## Introduction

Recently, the use of e-cigarettes has become popular as an alternative method to conventional tobacco cigarettes among adults (1). These battery-operated devices release heated liquid containing nicotine, glycerol, flavorings, and other chemical substances in the form of aerosol which is then inhaled into the lungs (1, 2). The prevalence of e-cigarettes has been rapidly increasing among young adults worldwide, influenced by media and industry companies’ promotion. This promotion often creates a perception that e-cigarettes are less harmful than traditional cigarettes (3). Previous research has identified various reasons for using e-cigarettes, including easy availability, accessibility, cost, flavor variety, and smoking cessation (1). In Saudi Arabia, a previous study indicated that the prevalence of e-cigarettes is specifically correlated with highly educated males between the ages of 18 to 24, who smoke tobacco frequently (4). While some teen users of e-cigarettes believe that e-cigarettes are less dangerous than conventional cigarettes, others think that it depends on the dose or frequency of use. Some users believe that they can prevent negative consequences and quit smoking before becoming addicted, considering them to be less harmful (5).

The use of e-cigarettes has been associated with several negative consequences. A study conducted by the Center for Tobacco Research and Educational Center at the University of California, San Francisco, found that daily use of e-cigarettes doubles the risk of heart attack (1). Nicotine in e-cigarettes can increase the risk of heart problems like high blood pressure and hardening of the arteries (6). Moreover, e-cigarettes are harmful to human organs, particularly the respiratory system (5). Research on the respiratory system has shed light on the understanding of the lung-damaging ingredients in e-cigarettes. Toxicants (chemicals, heavy metals, and nanoparticles) and toxins (beta-glucans and endotoxins) have been found in e-cigarettes. These substances can lead to elevated reactive oxygen species (ROS), DNA damage, elevated chemokines and cytokines, inflammatory cell infiltration and activity, and altered cellular processes. Numerous factors, including age, sex, underlying medical conditions, e-cigarette device type, vaping frequency, and e-liquid composition, can influence health outcomes due to the complexity of lung inflammation and injury (7). A study by Flouris et al. (8) found that the FEV1/FVC ratio decreased significantly after short-term exposure to both e-cigarettes and combustible cigarettes. Additionally, e-cigarettes can be harmful to oral health due to the ingestion of high levels of nicotine and other harmful substances such as metals, tobacco-specific nitrosamines, aldehydes, and volatile organic compounds, which can adversely affect the oral microbiota and oral epithelial cells (1).

Moreover, it has been demonstrated that e-cigarette use among teenagers severely impairs brain development, resulting in cognitive and mental health issues such as depression, anxiety, violence, substance abuse, and suicidality (9). A new longitudinal investigation revealed a correlation between smoking and lower psychological well-being and a correlation between higher psychological well-being and a decreased risk of smoking. Therefore, it is likely that e-cigarette use can both cause and result in poor mental health (10).

Participation in meaningful occupations is essential for individuals of all ages and abilities, as it improves their quality of life, and forms the core of occupational therapy (OT). Occupations include activities of daily living (ADL), instrumental activities of daily living (IADLs), education, play, work, social participation, rest/sleep, health management, and leisure (11). Previous studies have shown that e-cigarette use can significantly decrease both the quality and duration of sleep (12), as well as negatively impact academic or work performance (13), financial management (14), and oral hygiene (15). Additionally, e-cigarette use has been associated with the development of eating disorders (16), and a decreased likelihood of participating in leisure activities, such as sports (17). These previous findings suggest that e-cigarette use can have negative impacts on multiple aspects of an individual’s physical and mental health, as well as their overall quality of life.

Therefore, this study aims to explore the associations between e-cigarette use, psychological distress, and participation in daily activities among adults in Riyadh, Saudi Arabia. It also seeks to understand the demographic profile and usage patterns of e-cigarette smokers. The results of this study will have a significant impact on public health policies and programs that aim to decrease e-cigarette usage, mitigate its negative consequences, and promote healthier alternatives for quitting smoking.

## Materials and methods

### Study design and participants

An observational cross-sectional study was conducted by distributing an online survey to all individuals, both Saudi and non-Saudi, who are currently living in Riyadh city, using e-cigarettes, and aged above 18 years old. The minimum sample size needed for the study was determined to be 385 participants, based on an estimated sampling size at a 95% confidence level and a margin of error of 5%.

Data was collected between October 2022 and May 2023. Participants were excluded from the study if they did not live in Riyadh, were under 18 years old, or did not use e-cigarettes. The Institutional Review Board at Princess Nourah bint Abdulrahman University approved the study [HAP-01-R-059].

### Data collection

The self-reported questionnaire was distributed non-probably using convenience-sampling techniques to adults who use e-cigarettes in Riyadh through social media channels such as Twitter and WhatsApp, and their consent was obtained before participating in the study.

### Study tool

The structured questionnaire aims to examine the associations between e-cigarette use and psychological distress, as well as the perceived effects of electronic cigarette use on participation in activities of daily living (ADL) among Saudi adults in Riyadh. The first section of the questionnaire includes questions designed to assess sociodemographic characteristics, such as gender, age, academic level, chronic diseases, and employment status. This section is adapted from a previous study and is used with permission (4). The second section includes questions that evaluate the reasons, duration, amount, and status of using e-cigarettes, which are derived from a prior study with a similar goal to this one with permission (18). The third section, which is about perception on effects of electronic cigarette use on ADL, uses seven items, which include personal hygiene, financial management, health management, sleep quality, work performance, social participation, and participation in religious and spiritual activities, with responses ranging from strongly disagree = 1 to strongly agree = 5. A relevant score was created by summing up the individual coded items, and the overall score ranged between 7 and 35. Higher scores indicated perceived higher negative effects on ADL. Regarding psychological distress, data was collected using the Kessler 6 (K6) scale as a nonspecific mental health tool, which has six items measuring depression, hopelessness, everything was an effort, restlessness, nervousness, and worthlessness in the past 30 days (19). The responses were coded from 0 (none) to 4 (all). The psychological distress score was calculated by summing up these items, and the score ranged between 0 and 24. The validity of the questionnaire was assessed by five experts in the pulmonary medicine and rehabilitation field, who evaluated the questionnaire items on their linguistic, content, and relevance. They evaluated the questionnaire and suggested certain changes that the authors complied with. The experts verified the face and content validity of the questionnaire. The reliability was reached by pilot testing the questionnaire with 30 participants who matched the inclusion criteria, and they reported that the questionnaire was clear, understandable, and easy to answer. The internal consistency of the data was assessed, and a Cronbach’s coefficient alpha of 0.85 showed a high level of internal consistency.

### Statistical analysis

Statistical analysis was carried out using R Studio (R version 4.1.1). Frequencies and percentages were used to present categorical data, whereas continuous data were expressed as the median and interquartile range (IQR). The internal consistency of perceived effects on ADL and the psychological distress assessment was checked using a Cronbach’s alpha test. To investigate the independent factors associated with the outcomes, two multivariate linear regression models were constructed. The first model had the negative ADL score as the dependent variable, and the following independent variables were entered: demographic characteristics (gender, age, educational level, monthly income, marital status, employment status, and chronic conditions), history of tobacco smoking, and patterns of e-cigarette use (the reasons for using e-cigarettes, current use of e-cigarettes, time at which the first e-cigarette was used, symptoms encountered during e-cigarette use, and family history of e-cigarette use). For the second model, the psychological distress score was the dependent variable, and the same independent variables were used as in the first model, plus ADL score. The results of the regression analysis were expressed as beta coefficients and their respective 95% confidence intervals (95% CIs).

## Results

The demographic characteristics of the study sample were analyzed based on the responses of 396 e-cigarette smokers. Most participants were male (61.4%), single (84.1%), aged between 18 and 24 years (67.4%), and had obtained a college degree or higher (67.9%). Furthermore, 60.4% of the participants had a monthly income of less than 5,000 SAR, and 57.1% of them were unemployed. Chronic diseases were prevalent among 19.4% of the participants, with asthma being the most frequently reported (70.1%), followed by hypertension (26%), see **Table 1**.

**Table 1:** Demographic characteristics of e-cigarette smokers.

Parameter	Category	N (%)
Gender	Male	243 (61.4%)
	Female	153 (38.6%)
Age (year)	18 to 24	267 (67.4%)
	25 to 34	98 (24.7%)
	35 to 64	31 (7.8%)
Educational level	Less than high school	6 (1.5%)
	High school graduate	121 (30.6%)
	College graduate and above	269 (67.9%)
Monthly Income (SAR)	< 5,000	239 (60.4%)
	5,000 to 14,000	121 (30.6%)
	15,000 to 24,000	27 (6.8%)
	25,000 and above	9 (2.3%)
Marital Status	Single	333 (84.1%)
	Married	54 (13.6%)
	Widowed/Divorced	9 (2.3%)
Employment status	Employed	170 (42.9%)
	Unemployed	226 (57.1%)
Chronic disease	No	319 (80.6%)
	Yes	77 (19.4%)
Types of chronic diseases	Asthma	54 (70.1%)
	Arthritis	11 (14.3%)
	COPD	8 (10.4%)
	Hypertension	20 (26.0%)
	Lung cancer	2 (2.6%)
	Chronic kidney disease	1 (1.3%)

### Patterns of e-cigarettes use

**Figure 1:**
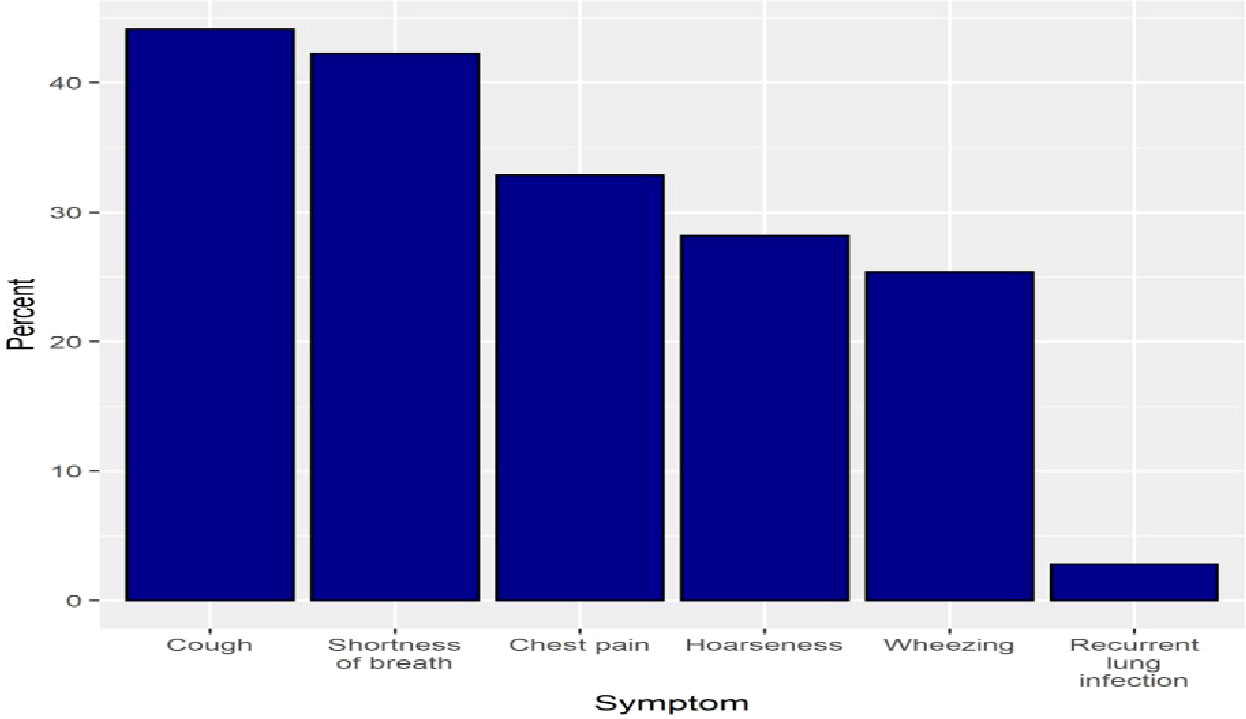
The percentage of symptoms encountered during the use of e-cigarettes.

Patterns of e-cigarette use were examined in the study. Less than half of the sample reported using e-cigarettes every day (45.7%). Additionally, 133 participants (33.6%) used e-cigarettes on some days, with 58.6% using e-cigarettes for one day and 41.4% using them for twenty days. The most common reasons for using e-cigarettes were to quit tobacco smoking (29.5%) and to use a safer way of smoking than tobacco cigarettes (16.2%). More than half of the respondents (56.3%) reported smoking their first e-cigarette within 30 minutes of waking up (**Table 2**). The most frequently reported symptoms during e-cigarette use were cough (44.1%) and shortness of breath (42.3%), (**Figure 1**). Additionally, 56.3% of the participants reported having a family member who smoked e-cigarettes. Roughly one-third of the respondents had never smoked a tobacco cigarette, while 23.7% and 23.2% were active every-day and some-day smokers, respectively (**Table 2**).

**Table 2:** Patterns of e-cigarettes use.

Parameter	Category	N (%)
The reason for using an electronic cigarette	Wanted to quit smoking cigarettes	117 (29.5%)
	Wanted to replace smoking cigarettes some of the time	27 (6.8%)
	Wanted to smoke in places where cigarettes smoking is not allowed	23 (5.8%)
	Safer than tobacco cigarettes	64 (16.2%)
	Cheaper than tobacco cigarettes	35 (8.8%)
	Other reasons	130 (32.8%)
Do you use it now	Not at all	82 (20.7%)
	Somedays	133 (33.6%)
	Everyday	181 (45.7%)
The number of days of using e-cigarettes (in the past 30 days)*	Once	78 (58.6%)
	Twenty or more days	55 (41.4%)
When do you smoke your first cigarette after been awake	More than 30 minutes	223 (56.3%)
	5 to 30 minutes	88 (22.2%)
	Less than 5 minutes	85 (21.5%)
Have any e-cigarettes smoker in the family	No	173 (43.7%)
	Yes, before	162 (40.9%)
	Yes, after	61 (15.4%)
Your smoking tobacco cigarettes status	Never smoker: Smoked <100 cigarettes in your lifetime	130 (32.8%)
	Former smoker: Smoked >100 cigarettes in your lifetime	80 (20.2%)
	Current someday smoker	92 (23.2%)
	Current every day smoker	94 (23.7%)

Categorical measures: n (%).

### Characteristics of distress and the activities of daily living

**Figure 2:**
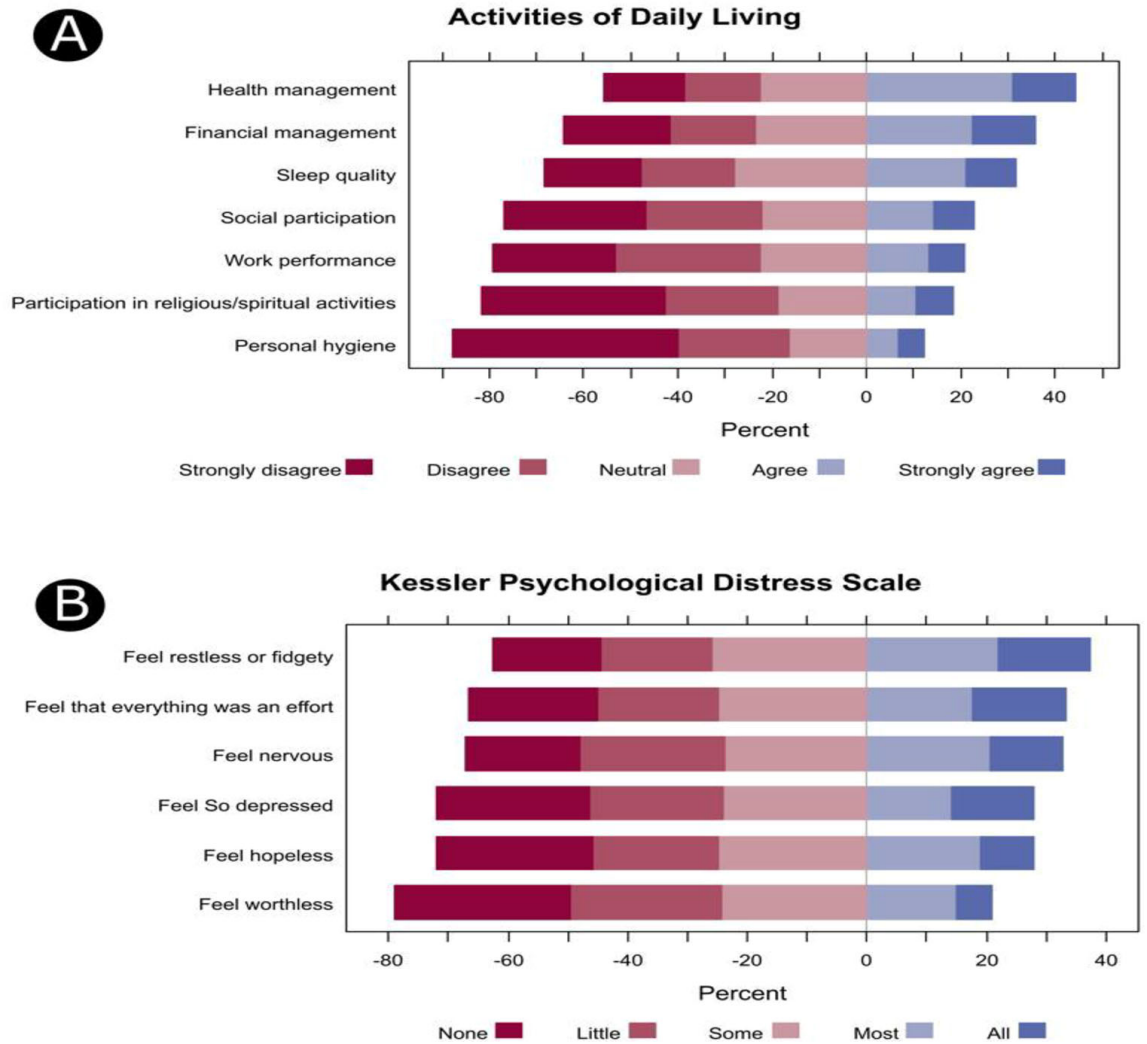
The percentage of participants’ responses regarding the perceived effects on the activities of daily living **(A)** and psychological distress items during the past 30 days **(B)**.

The perceived effect of e-cigarettes use on activities of daily living was assessed using a questionnaire with a maximum score of 35. The median score (IQR) was 18.0 (12.8 to 23.0), with excellent internal consistency reliability (alpha = 0.898 based on 7 items). Health management activities such as physical fitness (44.5%), financial management (35.9%), and sleep quality (31.6%) were the most frequently reported negatively affected daily activities during e-cigarette use (**Figure 2A**).

**Figure 3:**
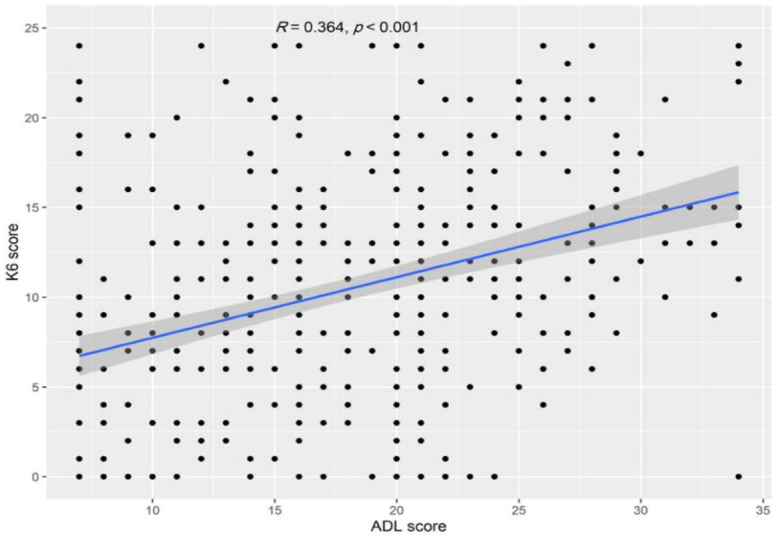
A scatter plot showing the correlation between ADL and K6 scores.

The psychological distress of participants was evaluated using the K6 scale, which has a maximum score of 24. The median (IQR) K6 score was 11.0 (6.0 to 15.0), and the internal consistency reliability was excellent (alpha = 0.912 based on 6 items). Participants frequently reported feeling restless or fidgety (37.4%), that everything was an effort (33.4%), and being nervous (32.8%) (**Figure 2B**). Correlation analysis showed a significant positive correlation between the ADL and K6 scores (Spearman’s r = 0.364, *p* < 0.001, **Figure 3**).

### Predictors of perceived effects on activities of daily living and psychological distress

The results of the multivariate regression analysis for predictors of perceived negative effects on activities of daily living and psychological distress are presented in detail in the = (**Supplementary Table S1**, **S2**, respectively), and the significantly associated independent factors are summarized in **Table 3**. The analysis revealed that perceived negative effects on activities of daily living were independently associated with having a history of COPD (beta = 0.35, 95% CI, 0.04 to 0.67, *p* = 0.029), experiencing cough (beta = 0.12, 95% CI, 0.02 to 0.22, *p* = 0.023), and shortness of breath (beta = 0.12, 95% CI, 0.01 to 0.23, *p* = 0.027) during e-cigarette use. Conversely, participants who perceived e-cigarettes as safer than tobacco cigarettes (beta = -0.17, 95% CI, -0.31 to -0.03, *p* = 0.021) and had other reasons for using e-cigarettes (beta = -0.18, 95% CI, -0.31 to -0.05, *p* = 0.006) were less likely to report negative effects on activities of daily living.

**Table 3:** Results of the linear regression analysis for the independent associated factors with perceived negative effects on activities of daily living and psychological distress.

Parameter	Category	Beta (95% CI)	*p*-value
The score of perceived negative effects on activities of daily living			
COPD	No	—	
	Yes	0.35 (0.04 to 0.67)	0.029
The reason for using an electronic cigarette	Wanted to quit smoking cigarettes	—	
	Wanted to replace smoking cigarettes some of the time	-0.11 (-0.29 to 0.08)	0.256
	Wanted to smoke in places where cigarettes smoking is not allowed	-0.08 (-0.28 to 0.12)	0.449
	Safer than tobacco cigarettes	-0.17 (-0.31 to -0.03)	0.021
	Cheaper than tobacco cigarettes	-0.11 (-0.27 to 0.06)	0.193
	Other reasons	-0.18 (-0.31 to -0.05)	0.006
Cough	No	—	
	Yes	0.12 (0.02 to 0.22)	0.023
Shortness of breath	No	—	
	Yes	0.12 (0.01 to 0.23)	0.027
The score of psychological distress			
Gender	Male	—	
	Female	3.25 (1.81 to 4.69)	<0.001
Age (year)	18 to 24	—	
	25 to 34	-1.93 (-3.72 to -0.15)	0.034
	35 to 64	-3.88 (-6.82 to -0.95)	0.010
Marital Status	Single	—	
	Married	0.76 (-1.41 to 2.93)	0.494
	Widowed/Divorced	4.52 (0.18 to 8.87)	0.041
Arthritis	No	—	
	Yes	4.38 (0.50 to 8.25)	0.027
Cough	No	—	
	Yes	1.55 (0.14 to 2.95)	0.031
Perceived negative effects on ADL	Number	0.26 (0.17 to 0.35)	<0.001

Regarding psychological distress, the multivariate regression model showed that participants aged 25 to 34 years (beta = -1.93, 95% CI, -3.72 to -0.15, *p* = 0.034) and 35 to 64 years (beta = -3.88, 95% CI, -6.82 to -0.95, *p* = 0.010) had lower psychological distress scores. Conversely, higher psychological distress scores were independently associated with being female (beta = 3.25, 95% CI, 1.81 to 4.69, *p* < 0.001), being widowed or divorced (beta = 4.52, 95% CI, 0.18 to 8.87, *p* = 0.041), having a history of arthritis (beta = 4.38, 95% CI, 0.50 to 8.25, *p* = 0.027), experiencing cough during e-cigarette use (beta = 1.55, 95% CI, 0.14 to 2.95, *p* = 0.031), and having higher scores of perceived negative effects on activities of daily living (beta = 0.26, 95% CI, 0.17 to 0.35, *p* < 0.001, **Table 3**).

## Discussion

This cross-sectional study aimed to investigate the correlation between e-cigarette use and psychological distress and the perceived effects of electronic cigarette use on participation in ADLs (activities of daily living) among adult Riyadh residents. Furthermore, it also aims to better understand the demographics and usage patterns of e-cigarette users. The majority of the participants reported that they do not suffer from any chronic diseases, while common respiratory symptoms experienced during smoking e-cigarettes were coughing and shortness of breath. Regarding the negative impacts associated with e-cigarettes use on ADL, participants frequently reported difficulties in managing their health, including physical performance, financial management, and sleep quality. Furthermore, psychological distress while using electronic cigarettes was often characterized by feeling restless, like everything was an effort, and anxiousness.

### Patterns of e-cigarette use

A greater understanding of the characteristics of e-cigarette usage would enable more targeted preventive and treatment actions (20). In this study, smoking E-cigarettes was more prevalent among males aged 18 to 24 who had completed college or higher education. Similarly, a prior study conducted in Saudi Arabia in 2019 found that the majority of e-cigarette smokers were highly educated males aged around 18-24 (4). In this study, the most frequently reported reasons for using e-cigarettes in this study were quitting tobacco use and finding a safer alternative to traditional cigarettes. Consistent with previous research, health issues were identified as a common reason for initiating e-cigarettes use (21). It has been reported that tobacco smokers utilize electronic cigarettes to quit smoking traditional cigarettes, with e-cigarettes serving as a substitute (13). The findings of this study indicate that smoking e-cigarettes is associated with a perceived negative effect on respiratory health, as participants reported coughing, shortness of breath, and chest pain while using electronic cigarettes. Chaffee et al, reported a significant association between e-cigarette use and high rates of respiratory symptoms, including bronchitis and shortness of breath, among young people in the United States (22). This study revealed that e-cigarettes use is common among smokers with chronic illnesses such as asthma and hypertension, and around half of the participants had a family member who used e-cigarettes. A previous study found a higher prevalence of e-cigarette use among patients with comorbid disorders such as asthma and cardiovascular disease. As they believed that electronic cigarettes could aid them quit smoking, reduce combustible cigarette consumption, and mitigate the adverse effects of smoking (23). A recent study also found that individuals with smokers in their families are more likely to become smokers than those without smokers in their families (24). Therefore, it can be inferred that people are influenced by their family members and mimic their smoking practices. These results highlight the necessity of involving peers and family in interventions, as they play a significant role in shaping attitudes and behaviors related to e-cigarette use.

### E-cigarettes and psychological distress

The findings of this study revealed a median K6 score of 11, indicating a high level of psychological stress among adult e-cigarette users. Several studies have investigated the association between e-cigarette use and psychological distress. Some studies indicate that e-cigarette use increases psychological distress, while others indicate that those who are already experiencing psychological distress are more likely to turn to e-cigarettes as a coping method. A previous study found that former smokers with a high level of psychological distress are more likely to use e-cigarettes (25). Another study reported that individuals with symptoms of anxiety and depression are at high risk of becoming e-cigarette users (26). Additionally, a different study demonstrated that adolescents who use electronic cigarettes exhibit a moderate level of emotional disorders, which is lower than combustible cigarette users but higher than non-smokers (25). Furthermore, a scoping review published in 2022, illustrated that the prevalence of e-cigarettes is increasing among adolescents between 10-21 years old, and their mental health is negatively affected while smoking electronic cigarettes (27). Consistent with previous studies, the majority of the individuals in this study reported feeling anxious, restless, and stressed out about everything. In addition, this study found a strong correlation between being female and experiencing severe psychological distress. This finding is supported by previous studies that reported that female e-cigarette users are more likely to experience negative effects on their mental health (27–29). Moreover, another study found that female adolescents who use e-cigarettes face higher risk of developing mental issues such as suicidal ideation and depression compared to males (27).

### E-cigarette use and participation in activities of daily living

Smoking increased the likelihood of experiencing difficulties in activities of daily living (ADL), regardless of cognitive functioning. However, it had a more negative impact on individuals who were already at a higher risk of cognitive impairment (30). A long-term follow-up study found that middle-aged smokers had a twice-as-higher risk of future impaired ADL compared to nonsmokers (31). Regarding the perceived detrimental effect associated with e-cigarette use on activities of daily living, several areas of daily living activity were affected, with the majority of study participants reporting a negative impact on their sleep quality. This is consistent with previous research findings that found a link between poor sleep quality and high e-cigarette use (32), as well as reports that most e-cigarette users e experience more sleep problems than non-smokers (33), and participants who used e-cigarettes had significantly shorter sleep durations (34). Financial management was another area affected by e-cigarette use, with participants reporting difficulties in managing their finances. This finding aligns with previous studies that have highlighted the financial burden associated with e-cigarette use which is more cost-effective than utilizing nicotine replacement medication (35). Health management was also negatively impacted in numerous areas, including physical fitness, as the participants reported In contrast, another study found that e-cigarette users are more likely to be physical activists when compared to cigarette smokers (17). Another study finding indicates that individuals who engage in fewer activities of daily living (ADLs), such as physical fitness, and have poor sleep quality, have a greater influence on their mental health. The study finding aligns with another study that found daily activities (ADL) may play a significant role in the link between poor sleep and mental stress. However, ADL only partially explains the relationship between poor sleep and psychological distress (36). The multivariate regression analysis for predictors of perceived negative effects of e-cigarette use on activities of daily living revealed that having a history of chronic obstructive pulmonary disease (COPD) and experiencing cough and shortness of breath were independently associated with lower ADL scores. Previous research data on the short-term impacts of e-cigarette usage shows that the vapor generated and inhaled by e-cigarette users has an adverse effect on airways and lung health of young people (37, 38). This may, therefore, impact their level of engagement in daily activities.

The results of this study contribute to the existing literature on the factors associated with e-cigarette use and its potential negative impacts on both physical and mental health. These findings have important implications for the development of health education interventions and effective smoking prevention policies in Saudi Arabia. Furthermore, the study highlights the need to raise awareness about the negative impact associated with e-cigarette use on the mental health of adults and their ability to participate in meaningful life activities.

## Conclusions

This exploratory study demonstrates that e-cigarettes are associated with negative impacts on various aspects of Saudi adults’ lives. The study’s findings indicate that highly educated individuals were the most common users of e-cigarettes, and the primary reason for utilizing e-cigarettes was to quit tobacco smoking. However, the use of e-cigarettes was associated with negative impacts on several activities of daily living (ADLs), including sleeping quality, financial management, and health management, such as physical activity. Additionally, e-cigarettes were found to play a critical role in Saudi adults’ mental health.

It is important to note that these findings of this exploratory study may not be generalizable to the wider population, as the study was conducted solely in Riyadh city, and convenience sampling was employed for participant selection. Therefore, future studies should be conducted across all cities in Saudi Arabia to increase the generalizability of the findings. Furthermore, the use of self-reported data and online platforms for data collection may have introduced a potential bias, potentially missing certain demographic groups that were intended to be included in the study. In future studies, alternative data collection methods and strategies should be considered to minimize this potential bias and ensure a more comprehensive representation of the target population. Further research is necessary to investigate the potential long-term impact of e-cigarette use on various aspects of individuals’ lives. Additionally, effective interventions must be developed to mitigate any negative impacts of e-cigarette use.

## Data availability statement

The authors will provide the raw data used to support the conclusions of this article upon request.

## Ethics statement

The studies involving humans were approved by Princess Nourah bint Abdulrahman University Institutional Review Board. The studies were conducted in accordance with the local legislation and institutional requirements. The participants provided their written informed consent to participate in this study.

## Author contributions

FA: Conceptualization, Methodology, Writing – original draft, Writing – review & editing. BA: Data curation, Investigation, Methodology, Writing – original draft. RA: Data curation, Formal Analysis, Investigation, Validation, Writing – original draft. HA: Data curation, Methodology, Software, Writing – original draft. RA: Data curation, Investigation, Methodology, Writing – original draft. GA: Data curation, Formal Analysis, Investigation, Methodology, Validation, Writing – original draft. HA: Conceptualization, Formal Analysis, Methodology, Writing – original draft. MT: Conceptualization, Formal Analysis, Funding acquisition, Methodology, Project administration, Supervision, Validation, Writing – original draft, Writing – review & editing.

## Supplementary material

Supplementary Table 1 Results of the regression analysis for the predictors of perceived negative effects on the activities of daily living.

Supplementary Table 2 Results of the regression analysis for the risk factors for distress as indicated by the K6 scale.
