# Supplementary material for: E-cigarette use, psychological distress, and daily activity participation among adults in Riyadh
Source: Front Psychiatry. 2024 Apr 12;15:1362233. doi: 10.3389/fpsyt.2024.1362233 (PMC11045981; doi:10.3389/fpsyt.2024.1362233)
Supplement: Supplementary Table 1 — Results of the regression analysis for the predictors of perceived negative effects on the activities of daily living. [file Table_1.docx]

**Supplementary file 1.**

Table S1: Results of the regression analysis for the predictors of perceived negative effects on the activities of daily living..

| Parameter | Category | Beta (95% CI) | *p*-value |
| --- | --- | --- | --- |
| Gender | Male | — |  |
|  | Female | 0.02 (-0.09 to 0.12) | 0.737 |
| Age (year) | 18 to 24 | — |  |
|  | 25 to 34 | 0.02 (-0.11 to 0.15) | 0.744 |
|  | 35 to 64 | 0.07 (-0.14 to 0.29) | 0.513 |
| Educational level | Less than high school | — |  |
|  | High school graduate | 0.01 (-0.38 to 0.41) | 0.941 |
|  | College graduate and above | -0.01 (-0.40 to 0.38) | 0.943 |
| Monthly Income (SAR) | < 5,000 | — |  |
|  | 5,000 to 14,000 | -0.04 (-0.18 to 0.09) | 0.502 |
|  | 15,000 to 24,000 | -0.05 (-0.25 to 0.15) | 0.617 |
|  | 25,000 and above | -0.22 (-0.53 to 0.08) | 0.153 |
| Marital Status | Single | — |  |
|  | Married | 0.01 (-0.15 to 0.17) | 0.872 |
|  | Widowed/Divorced | -0.06 (-0.38 to 0.26) | 0.725 |
| Employment status | Employed | — |  |
|  | Unemployed | 0.03 (-0.10 to 0.16) | 0.670 |
| Asthma | No | — |  |
|  | Yes | 0.02 (-0.11 to 0.15) | 0.784 |
| Arthritis | No | — |  |
|  | Yes | 0.04 (-0.24 to 0.33) | 0.770 |
| COPD | No | — |  |
|  | Yes | 0.35 (0.04 to 0.67) | 0.029 |
| Hypertension | No | — |  |
|  | Yes | 0.07 (-0.14 to 0.29) | 0.497 |
| Lung cancer | No | — |  |
|  | Yes | 0.19 (-0.74 to 1.11) | 0.692 |
| Chronic kidney diseases | No | — |  |
|  | Yes | 0.20 (-1.17 to 1.57) | 0.769 |
| The reason for using an electronic cigarette | Wanted to quit smoking cigarettes | — |  |
|  | Wanted to replace smoking cigarettes some of the time | -0.11 (-0.29 to 0.08) | 0.256 |
|  | Wanted to smoke in places where cigarettes smoking is not allowed | -0.08 (-0.28 to 0.12) | 0.449 |
|  | Safer than tobacco cigarettes | -0.17 (-0.31 to -0.03) | 0.021 |
|  | Cheaper than tobacco cigarettes | -0.11 (-0.27 to 0.06) | 0.193 |
|  | Other reasons | -0.18 (-0.31 to -0.05) | 0.006 |
| Do you use it now | Not at all | — |  |
|  | Somedays | 0.03 (-0.09 to 0.16) | 0.587 |
|  | Everyday | -0.08 (-0.21 to 0.05) | 0.221 |
| Have any e-cigarettes smoker in the family | No | — |  |
|  | Yes, before | 0.05 (-0.05 to 0.14) | 0.345 |
|  | Yes, After | -0.03 (-0.15 to 0.10) | 0.689 |
| When do you smoke your first cigarette after been awake | More than 30 minutes | — |  |
|  | 5 to 30 minutes | 0.03 (-0.09 to 0.15) | 0.624 |
|  | Less than 5 minutes | 0.05 (-0.07 to 0.17) | 0.373 |
| Cough | No | — |  |
|  | Yes | 0.12 (0.02 to 0.22) | 0.023 |
| Chest pain | No | — |  |
|  | Yes | 0.11 (-0.01 to 0.24) | 0.083 |
| Shortness of breath | No | — |  |
|  | Yes | 0.12 (0.01 to 0.23) | 0.027 |
| Hoarseness | No | — |  |
|  | Yes | 0.04 (-0.08 to 0.17) | 0.488 |
| Recurrent lung infection | No | — |  |
|  | Yes | 0.20 (-0.19 to 0.60) | 0.311 |
| Wheezing | No | — |  |
|  | Yes | 0.02 (-0.11 to 0.16) | 0.747 |
| Your smoking tobacco cigarettes status | Never smoker: Smoked <100 cigarettes in your lifetime | — |  |
|  | Former smoker: Smoked >100 cigarettes in your lifetime | -0.08 (-0.22 to 0.06) | 0.288 |
|  | Current Someday smoker | 0.02 (-0.11 to 0.16) | 0.713 |
|  | Current Every day smoker | 0.01 (-0.13 to 0.14) | 0.933 |

.
