# Supplementary material for: E-cigarette use, psychological distress, and daily activity participation among adults in Riyadh
Source: Front Psychiatry. 2024 Apr 12;15:1362233. doi: 10.3389/fpsyt.2024.1362233 (PMC11045981; doi:10.3389/fpsyt.2024.1362233)
Supplement: Supplementary Table 2 — Results of the regression analysis for the risk factors for distress as indicated by the K6 scale. [file Table_2.docx]

**Supplementary file 2.**

Table S2: Results of the regression analysis for the risk factors for distress as indicated by the K6 scale.

| Parameter | Category | Beta (95% CI) | *p*-value |
| --- | --- | --- | --- |
| Gender | Male | — |  |
|  | Female | 3.25 (1.81 to 4.69) | <0.001 |
| Age (year) | 18 to 24 | — |  |
|  | 25 to 34 | -1.93 (-3.72 to -0.15) | 0.034 |
|  | 35 to 64 | -3.88 (-6.82 to -0.95) | 0.010 |
| Educational level | Less than high school | — |  |
|  | High school graduate | 2.34 (-2.97 to 7.64) | 0.387 |
|  | College graduate and above | 3.39 (-1.87 to 8.65) | 0.206 |
| Monthly Income (SAR) | < 5,000 | — |  |
|  | 5,000 to 14,000 | -0.56 (-2.32 to 1.21) | 0.536 |
|  | 15,000 to 24,000 | -0.14 (-2.82 to 2.54) | 0.920 |
|  | 25,000 and above | 3.51 (-0.62 to 7.63) | 0.096 |
| Marital Status | Single | — |  |
|  | Married | 0.76 (-1.41 to 2.93) | 0.494 |
|  | Widowed/Divorced | 4.52 (0.18 to 8.87) | 0.041 |
| Employment status | Employed | — |  |
|  | Unemployed | -0.04 (-1.80 to 1.71) | 0.962 |
| Asthma | No | — |  |
|  | Yes | -1.46 (-3.26 to 0.35) | 0.113 |
| Arthritis | No | — |  |
|  | Yes | 4.38 (0.50 to 8.25) | 0.027 |
| COPD | No | — |  |
|  | Yes | 1.94 (-2.40 to 6.28) | 0.380 |
| Hypertension | No | — |  |
|  | Yes | 1.08 (-1.80 to 3.97) | 0.461 |
| Lung cancer | No | — |  |
|  | Yes | -7.63 (-20.1 to 4.83) | 0.229 |
| Chronic kidney diseases | No | — |  |
|  | Yes | 9.80 (-8.70 to 28.3) | 0.298 |
| The reason for using an electronic cigarette | Wanted to quit smoking cigarettes | — |  |
|  | Wanted to replace smoking cigarettes some of the time | 0.13 (-2.38 to 2.64) | 0.918 |
|  | Wanted to smoke in places where cigarettes smoking is not allowed | 1.69 (-1.00 to 4.38) | 0.218 |
|  | Safer than tobacco cigarettes | -1.37 (-3.29 to 0.54) | 0.159 |
|  | Cheaper than tobacco cigarettes | -0.38 (-2.58 to 1.83) | 0.738 |
|  | Other reasons | -0.27 (-2.01 to 1.48) | 0.763 |
| Do you use it now | Not at all | — |  |
|  | Somedays | 1.48 (-0.20 to 3.16) | 0.084 |
|  | Everyday | 1.46 (-0.28 to 3.20) | 0.100 |
| Have any e-cigarettes smoker in the family | No | — |  |
|  | Yes, before | 1.10 (-0.21 to 2.40) | 0.099 |
|  | Yes, After | 0.30 (-1.41 to 2.02) | 0.727 |
| When do you smoke your first cigarette after been awake | More than 30 minutes | — |  |
|  | 5 to 30 minutes | -0.60 (-2.20 to 1.01) | 0.463 |
|  | Less than 5 minutes | 1.18 (-0.45 to 2.80) | 0.156 |
| Cough | No | — |  |
|  | Yes | 1.55 (0.14 to 2.95) | 0.031 |
| Chest pain | No | — |  |
|  | Yes | -0.49 (-2.20 to 1.22) | 0.570 |
| Shortness of breath | No | — |  |
|  | Yes | 0.88 (-0.61 to 2.36) | 0.246 |
| Hoarseness | No | — |  |
|  | Yes | -0.13 (-1.83 to 1.57) | 0.879 |
| Recurrent lung infection | No | — |  |
|  | Yes | 1.93 (-3.40 to 7.27) | 0.476 |
| Wheezing | No | — |  |
|  | Yes | 1.70 (-0.11 to 3.50) | 0.065 |
| Your smoking tobacco cigarettes status | Never smoker: Smoked <100 cigarettes in your lifetime | — |  |
|  | Former smoker: Smoked >100 cigarettes in your lifetime | 0.01 (-1.89 to 1.91) | 0.990 |
|  | Current Someday smoker | 0.70 (-1.07 to 2.47) | 0.436 |
|  | Current Every day smoker | 1.36 (-0.47 to 3.20) | 0.145 |
| ADL | Number | 0.26 (0.17 to 0.35) | <0.001 |
